# RNA structure: implications in viral infections and neurodegenerative diseases

**DOI:** 10.1007/s44307-024-00010-2

**Published:** 2024-02-02

**Authors:** Suiru Lu, Yongkang Tang, Shaozhen Yin, Lei Sun

**Affiliations:** 1 Pingyuan Laboratory, Xinxiang, Henan, 453007 China; 2 Shandong Provincial Key Laboratory of Animal Cell and Developmental Biology, School of Life Sciences, Shandong University, Qingdao, 266237 China; 3 Taishan College, Shandong University, Qingdao, 266237 China; 4 State Key Laboratory of Microbial Technology, Shandong University, Qingdao, 266237 China

**Keywords:** RNA structure probing, RNA secondary structure, Post-transcription regulation, Human diseases

## Abstract

RNA is an intermediary between DNA and protein, a catalyzer of biochemical reactions, and a regulator of genes and transcripts. RNA structures are essential for complicated functions. Recent years have witnessed rapid advancements in RNA secondary structure probing techniques. These technological strides provided comprehensive insights into RNA structures, which significantly contributed to our understanding of diverse cellular regulatory processes, including gene regulation, epigenetic regulation, and post-transactional regulation. Meanwhile, they have facilitated the creation of therapeutic tools for tackling human diseases. Despite their therapeutic applications, RNA structure probing methods also offer a promising avenue for exploring the mechanisms of human diseases, potentially providing the key to overcoming existing research constraints and obtaining the in-depth information necessary for a deeper understanding of disease mechanisms.

## Introduction

RNA was discovered over a century ago, igniting intensive investigations into its biochemical properties. Over time, scientists unveiled the intricate connections between RNA and DNA, shedding light on RNA's pivotal role in the complicated process of protein synthesis (Brenner 1961; Crick 1970). Despite its primary role in polypeptide chain encoding, the improvements in molecular biology tools and the availability of sequencing technology led to the discovery of a wide variety of non-coding RNAs and associated phenomena, such as X Inactive Specific Transcript (Xist) and X chromosome inactivation (Lyon 1972). Concurrently, another wave is emerging with the revelation that RNAs exhibit catalytic functions (Cech 1989; Kruger et al. 1982). Moreover, the discovery of RNA viruses in the mid-twentieth century and the identification of human immunodeficiency virus (HIV)(Yilmaz 2001) led to significant advancements in investigating RNA viruses, demonstrating that RNA can act as a viable genetic material. The revelation that RNA, capable of carrying genetic information and possessing catalytic functions, could potentially be the genesis of life, led to the formulation of the "RNA world" hypothesis (Bartel and Szostak 1993; Noller et al. 1992).

In the last 20 years, RNA research has entered a new era, mainly focusing on its functions, disease-related mechanisms, and potential clinical applications. For example, scientists are interested in exploring RNA-interacting molecules, developing messenger RNA (mRNA) vaccines, and uncovering mechanisms that connect RNA with human diseases. One of the fundamental topics that underlie the functional study, mechanism discovery, and application development is RNA structure. Just like the structure–function relationship in proteins, the understanding of RNA structures would provide a more rational and precise approach to studying or engineering methods for RNA drug/vaccine development and human disease mechanism research.

Nowadays, many researchers in this field are trying to construct the structure–function maps for RNA molecules. Importantly, RNA molecules exhibit a notably high degree of structural variability, particularly in the absence of associated proteins. This diversity encompasses a broad spectrum of sequence-to-secondary structure relationships, making RNA research particularly challenging. Conventional techniques, including X-ray crystallography, nuclear magnetic resonance (NMR) spectroscopy, and cryo-electron microscopy (cryo-EM) frequently produce structures that deviate significantly from the actual biological configurations(Schroeder et al. 2004). Computational methodologies have sought to address this issue by employing thermodynamic calculations to derive the structure of RNA in its minimum free energy state (Hofacker 2003; Mathews et al. 2007). This approach, however, remains imperfect as the structure of RNA is regulated by multiple factors within the cellular environment.

Fortunately, there are now both more advanced experimental and computational methods available for RNA structure determination. People are now utilizing machine learning (Zhao et al. 2021) and deep learning (Fu et al. 2022; Sato et al. 2021; Singh et al. 2019; Wang et al. 2023; Townshend et al. 2021; Szikszai 2022) technologies for RNA structure prediction, which have been proven to be effective. By leveraging these technologies, researchers can analyze large amounts of RNA data and predict the structures of RNA molecules with greater accuracy. For the experimental methods, novel approaches that investigate RNA structures using enzymes and small molecule reactions were developed (Silverman et al. 2016). These methods enable researchers to extract information from the overall or specific RNA structures in vivo after serval technology iterations. These new methods, which have paved the way for studying the RNA structurome, not only facilitate the construction of structure–function connections in mechanism research but also provide solid information for developing and refining clinically applicable strategies.

In this review, we will delve into how RNA structures intricately participate in regulating intracellular functions, and, more importantly, how these functional insights can be applied to understand and manage human diseases. We will commence by briefly introducing pertinent structure probing techniques. Meanwhile, we will provide an overview of the significance of RNA structures concerning transcriptional and post-transcriptional regulations. Furthermore, we will discuss the state-of-the-art tools presently in development for potential applications in clinical therapeutics. Lastly, we will present two practical case studies illustrating the application of structure probing methods in exploring disease mechanisms.

## RNA structurome probing technologies and their applications in SARS-CoV-2

### Methods for probing RNA structurome

Over the last decade, substantial progress has been made in RNA chemical probing methods, enabling the comprehensive analysis of RNA structures in living cells at a genome-wide scale (Table 1). These RNA structure probing technologies can be broadly classified into two main categories (Piao et al. 2017).

**Table 1:** Summary of high-throughput approaches to probing RNA structure

Methods	Reagent	Features	Limitations	Refs
**Chemical probing – RT stop readout**				
SHAPE-seq 1.0/2.0	1M7, NMIA, BzCN	Unbiased probing four bases	SHAPE reagents cannot penetrate cell membranes	Lucks et al. 2011; Loughrey, et al. 2014)
DMS-seq	DMS	DMS can penetrate cell membranes which makes it suitable for probing in vivo RNA bases	Nucleotide bias—Only probing adenines and cytosines	Rouskin et al. 2014)
Structure-seq	DMS	Similar to DMS-Seq with additional DMSO controls	Nucleotide bias	Fang et al. 2015; Ding et al. 2014)
Structure-seq2	DMS	Additional biotinylated nucleotide eliminating purification steps to improve library building efficiency	Nucleotide bias	Ritchey et al. 2017)
Mod-seq	DMS	Similar to DMS-Seq with a particular focus on rRNA	Nucleotide bias	Talkish et al. 2014)
icSHAPE	NAI-N_3_	Probing genome-wide in vivo RNA structures without nucleotide bias Modified RNA molecules are enriched by biotin isolation to reduce background noises	Cannot generate direct base pairing information at modification sites Complicated library construction process	Spitale et al. 2015)
smartSHAPE	NAI-N_3_	Similar with icSHAPE but eliminated endogenous modification by RNaseI and biotin enrichment Control groups are not required Ultra-low input	Adopting the RT-stop strategy, cannot generate direct base pairing information at modification sites	Piao et al. 2022)
LASER-Seq	NAz	Light-generated probing Reactivity detected correlates with solvent accessibility	Nucleotide bias	Zinshteyn et al. 2019)
Lead-seq	Lead(II) irons	Probing single base, but modification patterns for probing sites are different from other probing signals	Lead(II) irons are toxic	Twittenhoff et al. 2020)
Keth-seq	N3-kethoxal	Probing G-quadruplexes	Only probing guanosine	Weng et al. 2020)
**Chemical probing – RT mutation readout**				
SHAPE-MaP	NAI,NAI-N_3_, 1M7, 1M6, NMIA	Single sequencing reads include more structure information than stop strategy	Higher sequencing depth Complicated calculation methods	Siegfried et al. 2014; Luo et al. 2021)
DMS-MaPseq	DMS	Similar to SHAPE-MaP	Nucleotide bias	Zubradt et al. 2017)
**RNA–RNA interaction –proximity ligation**				
PARIS	AMT + UVA	Direct capture of long-range in vivo RNA-RNA interactions RNA enrichment is conducted by 2D gel filtration UV light exposure	Efficiency of proximity ligation is low AMT crosslinking	Lu et al. 2016)
SPLASH	Biotinylated psoralen	Direct capture of in vivo RNA-RNA interactions	Efficiency of proximity ligation is low Psoralen crosslinking	Aw et al. 2016)
COMRADES	Psoralen	Direct capture of long-range in vivo RNA-RNA interactions Involved two enrichment steps	Efficiency of proximity ligation is low Psoralen crosslinking	Ziv et al. 2018)
RIC-seq	Formaldehyde	Capturing protein-mediated RNA-RNA interactions	Efficiency of proximity ligation is low Formaldehyde crosslinking	Cai et al. 2020)
CLASH	UVA	Direct capture of long-range RNA-RNA interactions	Efficiency of proximity ligation is low	Helwak et al. 2013)
MARIO	UVA	Direct capture of RNA-RNA interactions	RNA-RNA interactions detection has to be protein-mediated Efficiency of proximity ligation is low	Nguyen et al. 2016)
LIGR-seq	AMT	Direct capture of long-range RNA-RNA interactions	Efficiency of proximity ligation is low AMT crosslinking	Sharma et al. 2016)

*1M7* 1- methyl-7-nitroisatoic anhydride (1M7), *Amt* psoralen derivative 4’-aminomethyltrioxalen, *COMRADES* Crosslinking of matched RNAs and deep sequencing, *DMS* Dimethyl sulfide, *icSHAPE *in vivo SHAPE; LIGR-seq, Ligation of interacting RNA and high-throughput sequencing, *MARIO* Mapping RNA interactome *in viv*o, *SHAPE* Selective 2’ -Hydroxyl Acylation analyzed by Primer Extension), *Kethoxal* 1,1-dihydroxy-3-ethoxy-2-butanone, *LASER-Seq* Light Activated Structural Examination of RNA by high throughput sequencing, *NAI-N*_*3*_ Azide-modified 2-methylnicotinic acid imidazolide, *NAz* Nicotinoyl azide, *smartSHAPE* Small amount random RT icSHAPE

The first class is based on small-molecule modifications, which can provide insights into base-pairing possibilities (Wang et al. 2021). For instance, Dimethyl sulfide (DMS) and 2-(Azidomethyl) nicotinic acid imidazolide (NAI-N_3_) are two commonly used chemicals to probe single-stranded RNA (ssRNA) regions. The DMS can modify unpaired adenines (A) and cytosines (C) (Lightfoot and Hall 2014), while NAI-N_3_ can unbiasedly react with the unpaired ribose of unstructured nucleotides (Spitale et al. 2015). Consequently, this allows the identification of modified sites based on reverse transcription (RT), detecting stop sites of reverse transcriptase extension or chemically induced mutation positions.

The second class relies on crosslinking and proximity ligation (Kudla et al. 2020). Distinguishing itself from base modification techniques, crosslinking-based methodologies offer the advantage of directly capturing long-range RNA-RNA interactions. These processes involve crosslinking RNA within cells, followed by the extraction and fragmentation of RNA. Pairs of interacting fragments are then joined through ligation. The crucial information about RNA interactions is retained within these chimeric ligation products, which can be subsequently identified through sequencing and bioanalysis (Lu et al. 2016).

These aforementioned methods have enabled scientists to analyze the RNA secondary structure of various transcripts, including those in human, mouse cell lines, and certain viruses (Sun et al. 2019; Sun et al. 2021a; Lan et al. 2022). However, small chemical molecules, such as DMS, are unable to modify certain bases, and they also exhibit low efficiency in modifying bases with low solvent accessibility. Hence, certain studies are dedicated to refining the chemical molecules employed in structural probing techniques, for example, the development of 2-aminopyridine-3-carboxylic acid imidazolide (2A3) (Marinus et al. 2021), 2-methyl-3-furoic acid imidazolide (FAI) (Spitale et al. 2013), and NAI-N_3_ for in vivo RNA studies (Spitale et al. 2015). The RNA structural information obtained can subsequently be used for various applications, including but not limited to designing targeted drugs, developing vaccines, and searching for RNA-binding proteins. For detailed information on probing technologies, one could kindly refer to the following reviews (Wang et al. 2021; Qian et al. 2019; Spitale and Incarnato 2023).

### Application of RNA structurome probing methods in SARS-CoV-2 research

Severe acute respiratory syndrome coronavirus 2 (SARS-CoV-2) is responsible for the 2020 COVID-19 pandemic, and it belongs to the Betacoronavirus genus (Lu et al. 2020a). The genome of SARS-CoV-2 consists of single-stranded, positive-sense RNA and is known to be among the largest RNA viruses with approximately 30 kilobases (kb) in size (Jackson et al. 2022; Masters 2006). RNA genome itself, especially the RNA complex structures are integral to their functions and life cycle. Therefore, RNA structural elements and their potential for drug development against SARS-CoV-2 are actively researched to tackle virus-induced pathologies (Sun et al. 2021a). To achieve this, it is key to understand the RNA secondary structures of SARS-CoV-2.

**Fig. 1:**
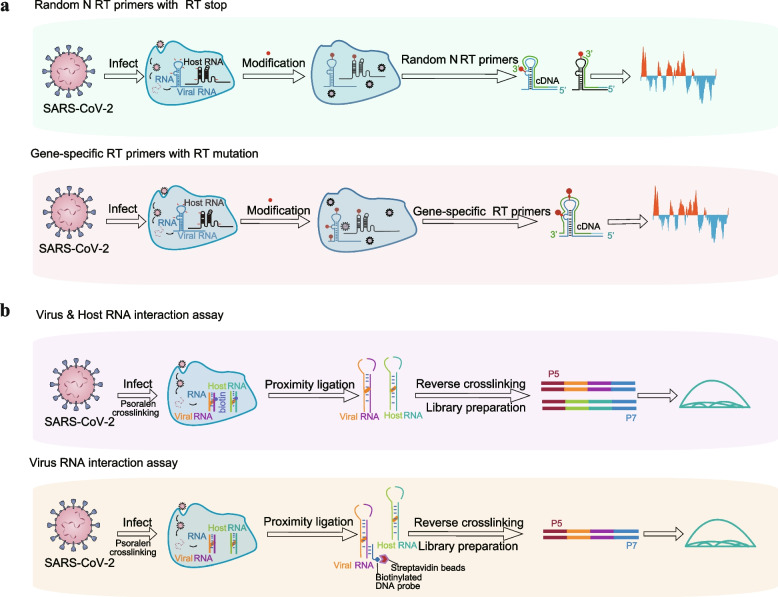
Illustration of the application of two classes of RNA structure probing methods in the context of SARS-CoV-2. **a,** Foot Printing-Based Probing Methods Workflow. Following SARS-CoV-2 infection, DMS or NAI-N_3_ is employed to modify the single-stranded RNA bases, including A and C, or the ribose of all four bases (A, U, C, G), respectively. These modifications result in distinct markers (red dots in the picture), which are subsequently converted into RT truncations or RT-induced mutations during reverse transcription. This process is facilitated by the use of either random N primers or gene-specific primers. The random N primer strategy provides information about both host and virus RNA structures, while the gene-specific primers exclusively yield information about the virus's RNA structure. The sites of RT truncation or RT mutations are then identified through sequencing and further analyzed using bioinformatics tools. **b,** Proximity Ligation-Based Probing Methods Workflow. After SARS-CoV-2 infection, RNAs are crosslinked by psoralen at regions where base pairing or interactions occur. The crosslinked RNAs are then fragmented. Proximity ligation is performed to connect viral RNA with other viral RNA molecules, as well as interactions between viral RNA and host RNA. Proximity ligation products can be enriched using gene-specific DNA probes. However, it's important to note that when enriching for virus RNA using this approach, it may lead to the specific acquisition of virus RNA interaction information while potentially losing information about host RNA interactions. Upon reversing the crosslinking, chimeric RNAs are generated, containing information about base pairing or interactions. This information can be retrieved from chimeric reads in sequencing libraries. The P5 and P7 shown in the figure represent two sequencing adapters ligated for sequencing during library preparations

Chemical probing is a critical strategy that has long been used for characterizing complex structures of RNA molecules. In recent studies, various models for the RNA secondary structure of the entire SARS-CoV-2 genome in human or monkey cells have been determined using RNA chemical probing techniques (Sun et al. 2021a; Huston et al. 2021). Research involving the chemical probing of the SARS-CoV-2 RNA genome can be broadly categorized into four approaches, which belong to two strategies mentioned in the previous section (Fig. 1). As demonstrated by the small-molecule modifications strategies shown in Fig. 1a, first, *in vivo* click selective 2-hydroxyl acylation and profiling experiment (icSHAPE) study conducted by Sun et al. used random N RT-primers to acquire structural information for both SARS-CoV-2 and the human transcriptome (Sun et al. 2021a). Similarly, the library construction could also be performed by adding an adaptor to the RNA before reverse transcription and using the adaptor sequence as the RT primer target. However, an additional genome fragmentation step needs to be included before adapter ligation (Spitale et al. 2015). Meanwhile, SHAPE with mutational profiling (SHAPE-MaP) and DMS mutational profiling with sequencing (DMS-MaPseq) obtained structural information specifically for the virus with specific primers designed for SARS-CoV-2 (Lan et al. 2022; Huston et al. 2021; Manfredonia et al. 2020).

One major concern for DMS or NAI-N_3_ probing methods is that they could not get long-range RNA-RNA interaction information. Fortunately, this can be tackled by utilizing the proximity ligation method to uncover interactions between viral RNA and other viral RNA molecules, as well as interactions between viral RNA and host RNA. With crosslinking and proximity ligation strategies (Fig. 1b), sequencing of psoralen cross-linked, ligated and selected hybrids (SPLASH) technique was used to acquire virus-virus, host-host, and virus-host RNA interactions (Zhang et al. 2021; Yang et al. 2021). Other methods involving an initial specific enrichment of SARS-CoV-2 RNA or virion are COMRADES (Ziv et al. 2020) and RNA in situ conformation sequencing (RIC-seq) (Cao et al. 2021), which highly improved the interested RNA probing depth. The development of these approaches has been instrumental in revealing the structure of hairpin-type pseudoknots, such as the frameshifting element (FSE) (Zhang et al. 2021; Ziv et al. 2020).

Thanks to the aforementioned RNA-structure probing technologies, numerous RNA structural elements have been identified within the SARS-CoV-2 genome (Sun et al. 2021a; Huston et al. 2021; Manfredonia et al. 2020). For example, multiple conserved RNA structural regions have been discovered, particularly those that are well-suited for designing antisense oligonucleotide (ASO) therapeutics (Manfredonia et al. 2020). ASOs can bind to conserved viral RNA regions and disrupt RNA structural functions to attenuate viral infection according to several published studies (Sun et al. 2021a; Manfredonia et al. 2020). For instance, specific ASOs were employed to disrupt SARS-CoV-2 structural elements within the open reading frame (ORF) ab (ORF1ab_6449 and ORF1ab_9456) and structural protein-coding regions (N_29502). These functional study results demonstrated the inhibited replication of SARS-CoV-2. To validate the structural significance in SARS-CoV-2 replication, mutations were specifically introduced to perturb base-pairing propensities in the ORF1ab_6449 structural element. As expected, viral RNA levels were drastically reduced, and this massive reduction could be partly rescued by tunning the displaced RNA structure back (Sun et al. 2021a). Apart from conserved regions, a wide range of techniques were adopted to unveil other dynamic RNA structures and interactions. Functional analysis, such as locked nucleic acids (LNAs), was employed to disrupt RNA structures to reveal the functional roles of certain RNA motifs within well-folded regions during the SARS-CoV-2 life cycle (Huston et al. 2021). Additionally, information about the secondary structure of RNA can be utilized to predict interactions between host proteins and the SARS-CoV-2 RNA genome. To give an example, the deep-learning tool Protein-RNA Interaction by Structure-informed Modeling using a deep neural network (PrismNet) was utilized to predict RNA-binding protein (RBP) and RNA interactions using icSHAPE scores (Sun et al. 2021b). Essentially, some of the interactions identified by PrismNet can serve as potential drug targets, thereby facilitating the repurposing of existing drugs to inhibit SARS-CoV-2 (Sun et al. 2021a).

Together, these developed tools and methods have made substantial contributions to our comprehension of the RNA secondary structure of SARS-CoV-2 and its interactions with host proteins and RNA. These discoveries offer hope for the development of effective therapeutics and our capacity to combat viral infections.

## RNA secondary structure-dependent regulation and applications

**Fig. 2:**
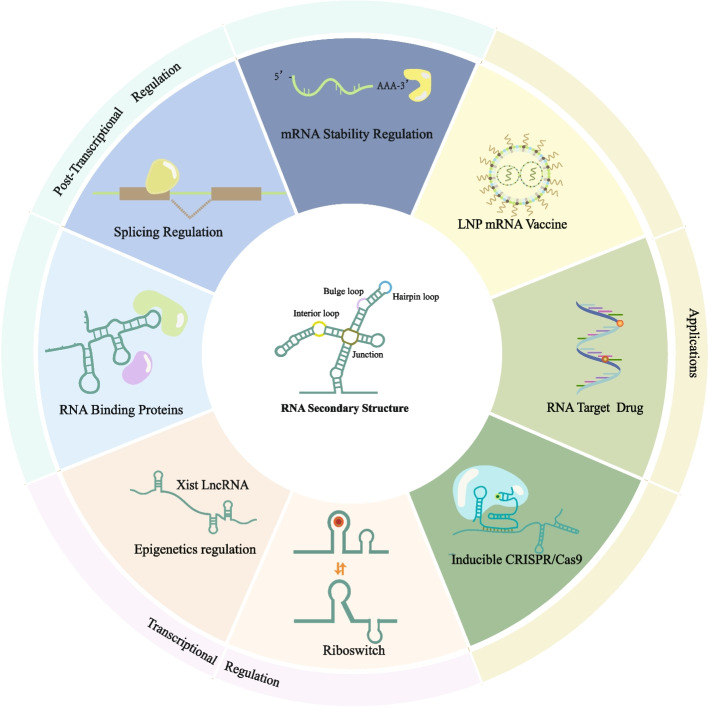
RNA secondary structure-dependent regulations and applications. The secondary structure of RNA serves a crucial function in both transcriptional and post-transcriptional regulation. These regulatory roles have a broad spectrum of practical applications. (i) Transcriptional regulation: epigenetic regulation can be modulated by the secondary structure of LncRNA. When riboswitches function as trans-acting elements, they have the capacity to influence transcription in various ways. (ii) Post-transcriptional regulation: post-transcriptional regulation activities, for example, RNA stability, RNA splicing, and RNA binding proteins, are closely related to RNA structure. (iii) Applications: RNA secondary structure can facilitate the research on therapeutic tools such as LNP mRNA vaccines, inducible CRISPR/Cas9 systems, and RNA-target drugs

The remarkable flexibility and diversity of RNA structures have established a fundamental basis for RNA’s pivotal role as a key regulatory mediator. Through the application of advanced RNA structure probing technologies, we have gained unprecedented insights into the secondary structural characteristics of a myriad of RNA molecules at a transcriptome-wide scale. Consequently, these revelations have contributed to our growing understanding of the integral roles that RNA structures play in both transcriptional and post-transcriptional regulation. As our comprehension of RNA secondary structure advances, there is a growing expectation that this knowledge will open new avenues for the treatment of human diseases. In this review, we explore crucial insights into the regulatory roles of RNA, particularly concerning its secondary structure, and its wide-ranging applications in the context of disease treatment (Fig. 2).

### RNA secondary structures are involved in transcriptional regulation

RNA assumes a critical role in the regulation of transcription via two primary mechanisms. First, it serves as an intermediary, facilitating interactions with various proteins. Second, it exerts control over the transcriptional state by modulating its structure. RNA molecules, characterized by intricate secondary structures, provide an ideal platform for accommodating multiple protein bindings, thereby bolstering the efficiency of these vital interactions (Sanchez de Groot, et al. 2019). Meanwhile, RNA has the capability to react to alterations in external stimuli and undergo conformational changes correspondingly.

#### LncRNA Xist plays a role in epigenetic regulation

Long non-coding RNA (lncRNA) represents a category of RNA molecules with a length exceeding 200 nucleotides. These molecules have been implicated in various pathological conditions, including prostate cancer (Takayama et al. 2020), hereditary diseases (Ramos et al. 2023; Cannavicci et al. 2020; Yang, et al. 2022), and neurodegenerative disease (Tan et al. 2023; Li and Wang 2023), as evidenced by numerous experiments. Nevertheless, the majority of lncRNAs remain unexplored in terms of their specific functions. Notably, Xist stands out as one of the most extensively studied among these lncRNAs and has been found to play a crucial role in the epigenetic inactivation of the X chromosome (Pacini et al. 2021).

The secondary structure of Xist has been successfully elucidated (Lu et al. 2016; Lu et al. 2020b; Fang et al. 2015; Smola et al. 2016). In 2015, Rui Fang et al*.* introduced an innovative method named "Targeted Structure-Seq" for the comprehensive investigation of RNA secondary structures, and was applied to probe the complete secondary structure of Xist (Fang et al. 2015). Their research led to an updated comprehension of the repeat A region and the discovery of a previously unidentified conserved element within the repeat C region. The conserved structure in the repeat A region is essential for gene repression and the disruption of the conserved elements in the C region can displace the Xist from chromatin. Researchers used SHAPE-Map, a method that can determine RNA secondary structure with single-nucleotide resolution, to investigate the complex structure of Xist and its interactions within the cellular environment. Also, they found that the E region's repeat structure can act as a binding platform for several protein cofactors, including CELF1, PTBP1, and HuR (Smola et al. 2016). These bonded proteins can regulate processes such as Xist splicing and RNA decay, which further regulate X chromosome deactivation.

Another technique that also provides near single-nucleotide resolution, known as psoralen analysis of RNA interactions and structures (PARIS), relies on reversible psoralen crosslinking. This innovative approach can identify long-range RNA-RNA interactions, thereby revealing higher-order RNA structures (Lu et al. 2016). They found that the inter-repeat duplex structure of A region can recruit SPEN. SPEN protein is a key epigenetic silencing protein that also carries an indispensable role in initiating X-chromosome inactivation.

In a separate study, a structure–function map of the Xist RNA–protein complex was created through the integration of fRIP-seq, eCLIP, and a combination of PARIS, icSHAPE, and conservation analysis (Lu et al. 2020b). During the research, they altered locations of the repeat A region in Xist and found that related proteins can nucleate correspondingly, which means that the A region is the center for ribonucleoprotein (RNP) assembly. They also identified that the modular structure of Xist can facilitate N^6^-methyladenosine (m^6^A) modification, which is proven to have various roles in gene expression regulations (Jiang et al. 2021).

These studies collectively indicate that Xist is a highly dynamic lncRNA characterized by several conserved structural domains, serving as a platform for interaction with numerous proteins (e.g. SPEN). These conserved structural elements underlie Xist's crucial roles in epigenetic inactivation of the X chromosome (Patil et al. 2016).

#### Riboswitch—RNA as a regulator to control transcription

Riboswitch represents a class of structured non-coding RNA elements that has the capacity to bind to small molecules. Upon binding, riboswitches undergo secondary structural changes that exert a direct impact on gene expressions (Bédard et al. 2020). Riboswitches typically comprise two distinct domains: a relatively conserved aptamer domain and an expression platform. Riboswitches are characterized by their acute responses, high sensitivity, and a broad array of available ligands or corresponding components. These features make them attractive candidates for potential therapeutic applications. As our understanding of structural and functional aspects of riboswitches grows, computational approaches, including rational or semi-rational design, can be used to discover natural and synthetic riboswitches with specificity, allowing researchers to target and regulate gene expression in normal and disease settings (Wu et al. 2023; Gong, et al. 2017). Nonetheless, due to the dynamic nature of RNA structure within living cells, designed riboswitches that perform effectively in vitro may not function as expected in vivo. Essentially, the regulation of riboswitches relies on the transitions between two relatively stable structural conformations (Bédard et al. 2020). This transition can be triggered by various factors such as small molecules, including metabolites, metal ions, and even other RNA molecules, offering substantial opportunities for exploitation and fine-tuning.

In nature, riboswitches generally serve as cis-acting regulatory elements, influencing the expression of downstream genes via RNA structural changes. A prominent instance of such regulatory elements is the Theophylline-dependent riboswitch (Nakahira et al. 2013). In this particular case, the gene subjected to regulation is positioned downstream of the riboswitch. As the concentration of theophylline attains a certain threshold, the riboswitch can activate the gene as required. This mode of action predominantly occurs in post-transcriptional regulation, encompassing processes such as translation regulation, splicing control, and mRNA degradation. In contrast, in the realm of transcriptional regulation, riboswitches frequently serve as trans-acting elements. For example, they may collaborate with the clustered regularly interspaced short palindromic repeats (CRISPR) system to facilitate gene editing when the respective effectors present (Cengic, et al. 2022), which we will discuss in the third part.

### Post-transcriptional regulation is highly dependent on RNA structure

Post-transcriptional regulation encompasses a diverse array of processes occurring after RNA transcription. These regulatory mechanisms include RNA localization, stabilization, splicing, and translation, among others. RNA structure plays a pivotal role in shaping specific processes within this intricate regulatory network, forming a fundamental aspect that directly influences various functions. In the following sections, we will elucidate several pivotal processes and delineate the role of RNA structure in each.

#### RNA stability regulation mainly associates with RNA structure in the 3’ end

Numerous intracellular processes demand precise coordination with the RNA life cycle, necessitating meticulous regulation of RNA abundance. This involves the precise control of RNA stabilization and degradation for the seamless operation of the cell. RNA decay encompasses multiple intricate pathways. The mRNA, for instance, can undergo either 3′ → 5′ decay (e.g., through the exosome pathway) or 5′ → 3′ decay (e.g., via the XRN1 pathway) (Garneau et al. 2007). The stability of RNA is contingent on various factors, including RNA modifications, sequence characteristics, RNA-binding proteins, and cellular states, among others. Multiple studies also showed that the balance between RNA decay and RNA stability can be influenced by RNA structure. Structured 3’ untranslated regions (UTRs) tend to undergo decay, while more flexible 3’ UTRs tend to remain stable (Fischer et al. 2020; Rasekhian et al. 2021). A potential RNA decay mechanism has been proposed involving two key proteins, UPF1 (an RNA-binding protein) and G3BP1 (associated with UPF1). The depletion of either protein leads to the accumulation of mRNAs with highly structured 3’ UTRs, underlying a structure-dependent mechanism of RNA regulation. Additionally, research revealed that the predominant factor influencing RNA stability is the base-pairing density in the 3' UTR (Fischer et al. 2020). The high density of base-pairing indicates proximity and readiness to initiate structure-mediated RNA decay (SRD).

The 3’ UTR of OX40 mRNA, as one of the examples of structural regulation of RNA stability, was initially characterized by Tants et al*.* They identified four distinct structural elements, namely the bulge, the ADE, the CDE, and the kinked-helical terminus (Tants et al. 2022). Interestingly, rather than the sequence features of the ADE and CDE elements, the key mRNA stability regulator Roquin tends to recognize the stem-loop structure (Braun et al. 2018). Moreover, Goodarzi et al*.* developed TEISER (Tool for Eliciting Informative Structural Elements in RNA) and have identified 8 structural elements that are crucial in the stability regulation of mammalian mRNAs (Goodarzi et al. 2012). Among these eight structural elements, sRSM1 plays a pivotal role in global mRNA regulation by interacting with the binding protein HNRPA2B1. Essentially, the stability of RNA is a crucial factor to be addressed in the context of RNA vaccine development, which we will explore further in the next part.

#### Splicing regulation

Pre-mRNA contains both exons and introns, and splicing is the pivotal process involving the removal of introns and the ligation of exons. Multiple arrangements of exons on the same mRNA give rise to alternative splicing (Marasco and Kornblihtt 2023). In this process, the RNA, acting as a ribozyme, assumes a significant role (Wilkinson et al. 2020), which underscores the influence of RNA structure on this catalytic process.

A substantial body of evidence underscores the critical role of RNA structure in the regulation of splicing. For instance, one study identified distinct asymmetric RNA secondary structures at exon-exon junctions in both denatured and naturally deproteinized transcripts. Specifically, the 5' exon ends tend to be more accessible, while the 3' exon ends are more structured. This implies that certain structures may be involved in the splicing process (Wan et al. 2014). Another investigation established a robust connection between splice site GC content and splice site usage, suggesting that compact secondary structures formed by high GC content may facilitate alternative splicing (Zhang et al. 2011). Furthermore, competing RNA secondary structures represent a significant mechanism in mutually exclusive splicing. As a widely applicable mechanism proposed in 2011 suggests, inter-intronic RNA pairing ensures the selection of only one exon in mutually exclusive splicing (Yang et al. 2011). Additionally, Hou et al*.* discovered that competing RNA pairs facilitate both cis-splicing and trans-splicing in Dscam1, leading to the generation of diverse isoforms (Hou, et al. 2022). The subsequent section will delve into the discussion of how unexpected splicing events can give rise to human diseases.

#### RNA Binding Protein

RBPs are the primary agents through which RNA regulates cellular states. It is also noteworthy that RBPs represent important drug-targeted sites (Prasad et al. 2022). Traditionally, the characterization of protein binding has often relied on sequence information. However, the importance of structural information yielding more precise insights for research has gradually gained traction. On the one hand, these RBPs interact with RNA, regulating functions such as RNA splicing, RNA stability and decay, and RNA virus activities (see related part). On the other hand, highly structured RNAs provide platforms for the interplay between proteins that work collectively to carry out certain functions, such as epigenetic regulation.

There is increasing evidence to suggest that interactions between RBPs and RNAs are structural-dependent. For example, Roquin recognizes the particular RNA stem-loop structure to regulate the mRNA stability (Tants et al. 2022). Additionally, researchers have discovered that the accuracy of deep learning models for RBP prediction can be significantly improved by incorporating RNA secondary structure information during training. This improvement has been demonstrated by PrismNet (Xu et al. 2023) and high-throughput dynamic cellular RNA-binding event identification using deep neural network (HDRNet) (Zhu et al. 2023). In another study, transfer learning was employed by integrating RNA secondary structure information to predict potential protein target sites, resulting in increased accuracy of the prediction results (Vaculík, et al. 2023).

Most importantly, RNA secondary structure can regulate virus activities through RBP. To give an example, SND1 (Staphylococcal Nuclease And Tudor Domain Containing 1) is proven to have higher affinity with RNA hairpin structure compared with single strand RNA (Xu et al. 2023). In another study, it was reported that the host SND1 protein can recognize the antisense RNA of SARS-CoV-2 and can regulate its replication in the host cell (Schmidt et al. 2023). The lower expression of SND1 is corresponded to reduced viral replication.

There are several experimental methods available for studying protein-RNA interactions in vivo, including RNA immunoprecipitation (RIP) (Gilbert and Svejstrup 2006) and a technique that combines crosslinking and immunoprecipitation (CLIP) and sequencing (Nostrand et al. 2016; Hafner et al. 2010; Licatalosi et al. 2008). Furthermore, many researchers also employ machine learning or deep learning methods to integrate experimental data for predicting RBP binding (Sun et al. 2021b; Xu et al. 2023; Zhu et al. 2023; Vaculík, et al. 2023; Ghanbari and Ohler 2020).

### From regulation to application: Insights of RNA secondary structure facilitates development of therapeutic tools for human diseases

RNA plays a pivotal role in a wide spectrum of biological processes and has been implicated in the development of a broad array of diseases. RNA exhibits a vast array of structures and engages in interactions with numerous proteins. When these interactions go awry, diseases are initiated. As our comprehension of RNA, particularly its structural intricacies continue to advance, researchers start to explore the potential applications of RNA in clinical disease management.

The following section provides an overview of how our understanding of RNA secondary structure has contributed to the development of clinical therapeutic tools for human diseases. We will focus on three prominent examples: mRNA vaccines, the CRISPR/Cas9 system, and RNA-targeted drugs.

#### Lipid nanoparticles (LNP)-mRNA vaccines

Traditional vaccines often entail a lengthy and time-consuming development process, whereas mRNA vaccines can be developed with greater expediency (Gote, et al. 2023). Notably, mRNA vaccines offer enhanced safety and cost-effectiveness with superior efficacy in comparison to conventional vaccines (Zhang et al. 2023a).

Research on mRNA vaccines can be categorized into two primary domains: vaccine development and the creation of effective vaccine delivery systems. The former is primarily concerned with optimizing the desired effects of mRNA, while the latter focuses on enabling RNA to target specific areas within the body. Collectively, mRNA vaccine stability and the development of efficient delivery systems are the current hindrances to the advancement of mRNA vaccines. A comprehensive overview of the general progress in mRNA vaccine development is available elsewhere (Gote, et al. 2023). Expanding upon our previous assertion that the structural component located at the UTR ends of the mRNA enhances RNA stability, we will now delve into how this knowledge is applied in the actual production of vaccines.

One study has enhanced the immunogenicity and protective efficacy of the SARS-CoV-2 vaccine in non-human primates by optimizing the unmodified RNA's 5' UTR (Gebre et al. 2022). They discovered that the structural modifications in the 5' UTR of CV2CoV, a second-generation mRNA vaccine with an optimized non-coding region, significantly contribute to robust protection. Thus, optimizing the 5' UTR becomes imperative, given its association with protein translation efficiency (Ryczek et al. 2023). Zhang et al*.* have proposed an algorithm, LinearDesign, designed to optimize RNA secondary structure, stability, and codon usage for mRNA vaccines in less than 16 min. This innovation not only reduces labor costs but also enhances the effectiveness of mRNA vaccines (Zhang et al. 2023b).

#### Inducible CRISPR/Cas9 system based on riboswitch

The CRISPR/Cas gene-editing technology, which initially originated in the immune systems of prokaryotes, has emerged as a groundbreaking tool (Barrangou et al. 2007; Brouns et al. 2008; Liu et al. 2022). Among the best-known Cas proteins consisting of Cas9 (type II), Cas12 (type V), and Cas13 (type VI), CRISPR/Cas9 is the most extensively utilized system. An overview of the advances and challenges in the use of CRISPR/Cas9 is available elsewhere (Li et al. 2023). In this context, we will discuss the inducible CRISPR/Cas9 system, which relies on riboswitches.

The inducible CRISPR/Cas9 system represents a precision-enhanced approach with reduced off-target effects, activated by external or internal signals. There are three primary methods to achieve inducibility in CRISPR systems. The first approach involves controlling the timing of guide RNA (gRNA) or Cas nuclease expression, ensuring their release at specific times to initiate CRISPR. The second method entails engineering nucleases, including the regulation of protein conformation. The last method involves manipulating the gRNA, which can be further categorized into two similar strategies reminiscent of riboswitches. One approach is to incorporate a non-functional ribozyme at the 5' end of gRNA, while the other method involves adding a sequence that prevents gRNA targeting. When a signaling molecule, such as a small molecule drug, binds to the added sequence, it induces a change in secondary structure, consequently, releasing the CRISPR/Cas9 system or exposing the gRNA sequence.

In 2016, Liu et al*.* introduced a CRISPR/Cas9-based signal conductor by incorporating a signal-responsive aptamer. They utilized this conductor as a 'switch' to redirect nucleophosmin (NPM)-mediated signaling from promoting proliferation to inducing quiescence in bladder cancer (Liu et al. 2016). Another study employed internal signals, specifically microRNAs (miRNAs), to trigger the CRISPR/Cas9 system when they are released and bind to the binding site in the pre-single guide RNA (pre-sgRNA) (Wang et al. 2019). The pre-sgRNA is non-functional, and upon miRNA binding to the pre-sgRNA's binding site, the pre-sgRNA undergoes cleavage, releasing mature and functional sgRNA molecules. The design of these aforementioned riboswitches relies heavily on RNA secondary structures.

#### RNA-targeted drug

As mentioned earlier, RNA's involvement in numerous diseases makes it a vital target for therapeutic interventions. Drug delivery systems often face challenges in traversing the blood–brain barrier, while the practical application of gene editing tools is hindered by collateral activities and off-target effects. In light of these challenges, small-molecule drugs, known for their ability to readily penetrate the blood–brain barrier, emerge as a compelling choice.

Accurate RNA structure models are crucial in the development of RNA-targeted drugs. Presently, most drug development efforts are grounded in the secondary structure of RNA to identify functional elements suitable for drug design. For instance, Sarah et al*.* employed SHAPE-Map to explore interactions between small molecules and RNAs (Martin et al. 2019). Essentially, the utilization of SHAPE allows us to acquire in vivo RNA secondary structure models better suited for the design of small molecule drugs (Martin et al. 2019; Smola et al. 2015).

## RNA Structure Probing Methods Aids in the Discovery of Human Disease Mechanisms

**Fig. 3:**
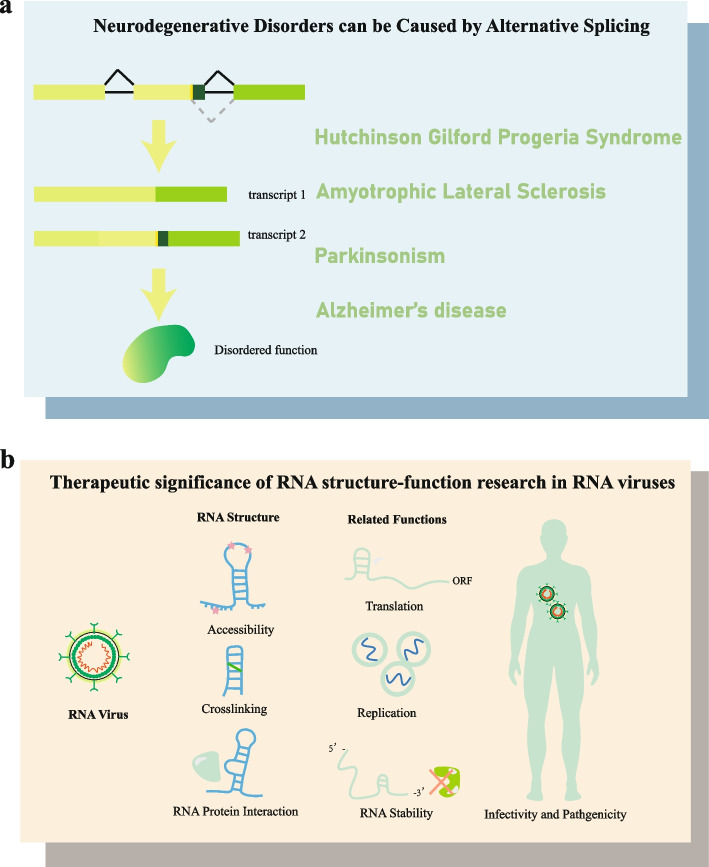
RNA structure probing methods aid the discovery of human disease mechanisms. **a** Neurodegenerative disorders can be caused by alternative splicing, and RNA structural information can provide insights into the research of the mechanisms. **b** Therapeutic significance of RNA structure–function research in RNA viruses. Various RNA structure probing methods are now accessible, facilitating a deeper comprehension of RNA virus infectivity and pathogenicity

Numerous disease mechanisms remain the subject of ongoing exploration, limited by the current extent of our knowledge. As our understanding of RNA continues to expand, its role in various diseases becomes increasingly evident. As highlighted earlier, RNA structure plays a pivotal role in multiple regulatory processes. It is now clear that a deeper comprehension of disease mechanisms often arises when RNA structure is taken into account. RNA structure exerts regulatory control over alternative splicing (as discussed in Part III), and it is noteworthy that at least 10% of diseases can be attributed to erroneous alternative splicing (Krawczak et al. 2007). Additionally, RNA structure has a substantial impact on the behavior of disease-causing viruses. In the sections that follow, we will elucidate how RNA structure probing technology facilitates the exploration of these two critical mechanisms (Fig. 3).

### Neurodegenerative disorders can be caused by alternative splicing

Changes in the gene, such as point mutations, insertions, or deletions, can result in altered RNA secondary structures. As illustrated in the third part, changes in RNA secondary structure affect cellular functions via transcriptional and post-transcriptional regulations, and potentially lead to human diseases. In most cases, human diseases are triggered by abnormal RNA secondary structures that cause unusual alternative splicing or RBP interactions. Here, we will focus on how RNA probing methods can help explore the mechanisms of diseases that are caused by alternative splicing.

Diseases such as Hutchinson-Gilford Progeria Syndrome (HGPS) (Rahman et al. 2021), Amyotrophic Lateral Sclerosis (ALS) (Feldman et al. 2022; Masrori and Damme 2020), Parkinsonism (Jakubauskienė and Kanopka 2021), Alzheimer’s disease (AD) (Waheed et al. 2023), and various types of cancers, such as bladder, brain, lung, gastric, and prostate cancers (Xie et al. 2019; Babic et al. 2013; Sheng et al. 2018; Ailiken et al. 2020; Bradley and Anczuków 2023), are closely linked to alternative splicing of RNA (Fig. 3a). Alternative splicing generates new transcripts that encode proteins with altered structures, prohibiting them from performing normal functions (Rahman et al. 2021; Waheed et al. 2023).

HGPS is a rare genetic disorder characterized by accelerated aging in affected patients (Rahman et al. 2021). This condition arises from aberrant alternative splicing of exon 11 of the *LMNA* gene. The presence of a mutation in the *LMNA* gene leads to the replacement of the original 5' splice site with an internal site. This, in turn, results in the production of a structurally disordered protein (Rahman et al. 2021; Lopez-Mejia et al. 2011). To investigate this internal site selection mechanism, Asaf et al*.* employed SHAPE-Map to examine the structural features of both the wild-type and C1824U mutant transcripts (Shilo et al. 2019). The results revealed striking similarities in the overall structures of the two variants. However, upon the disruption of the surrounding structural elements, there was an increased utilization of the alternative 5' splice site, suggesting that structural information holds more significance than sequence information in the pathogenesis of HGPS. This example underscores the pivotal role of RNA structural information, particularly at the 5' splice site, in determining the selection of alternative splicing sites. Various supporting factors, including regulatory proteins, also contribute to this process. For a comprehensive history of understanding the 5' splice site, a detailed review is available elsewhere (Roca et al. 2013).

Another example is Alzheimer’s disease (AD), which is associated with the dysregulation of the tau protein variants ratio (4R:3R ratio) (encoded by the *MART* gene). The role of RNA secondary structure in the splicing regulation of the *MART* gene has been investigated (Strang et al. 2019; Kumar, et al. 2022). In one of the studies, DMS-Map was used to examine the structures of pre-mRNA and mature *MART-encoded* mRNA, and data were collected for predicting alternative splicing outcomes (Kumar, et al. 2022). They found that the mutations can change the structural ensembles in the exon10-intron10 junction, differentiating alternative splicing events. Within normal human brain tissue, the 4R:3R ratio is approximately 1. When the structure of the 5' splicing site of exon10-intron10 is rather stabilized (stem-loop formation), exon10 tends to be skipped during alternative splicing and producing 3R isoform. When the stabilized structure of this junction region is shifted, the 5' splicing site becomes more accessible and gives rise to the 4R isoform. This result can be verified in previous research observations (Donahue et al. 2006), which found that mutations modifying the stem-loop structure at the exon–intron interface of exon10 ultimately led to the increased 3R mRNA isoform production. This study shows the integration of RNA structure probing method with computational methods to delve into the mechanisms of diseases.

These two specific diseases caused by unusual alternative splicing provide insights into how RNA secondary structure probing methods can be used to facilitate disease mechanism explorations. Moreover, these probing methods can also be combined with computational methods to predict disease outcomes.

### Therapeutic significance of RNA structure–function research in RNA viruses

RNA viruses belong to a category of viruses that utilize RNA as their genetic material (V'Kovski et al. 2021). Many RNA viruses can cause diseases in human bodies, and new disease-causing RNA viruses continue to be discovered. For instance, SARS-CoV-2 and Ebola viruses can cause severe acute respiratory syndrome (Hu et al. 2021) and Ebola Virus Disease (EVD) (Liu, et al. 2022) respectively. Also, Zika virus (ZIKV) is associated with infectious diseases, birth defects, and neurological disorders (Marbán-Castro et al. 2021). There are many other RNA viruses, such as HIV, hepacivirus, and influenza A virus, that can initiate human diseases and have caused global concern.

Despite the comparatively simple genomes of RNA viruses, their genetic material is remarkably compact and organized by intricate structural elements. As research has been well-demonstrated, RNA structure is closely related to its function. Numerous viral processes, including virus translation, RNA decay, and viral replication are intricately intertwined with RNA structure (Fig. 3b). These processes significantly impact viral infectivity and pathogenicity which warrant in-depth investigation.

Virus translation is vital for infectivity and pathogenicity, serving as the initial step to fulfill its role in host cells for positive-sense RNA viruses. According to research, translation efficiency is related to the frequency of single-stranded regions in SARS-CoV-2, highlighting the importance of RNA secondary structures in viral translation (Sun et al. 2021a). The internal ribosome entry site (IRES) is a class of structural elements located in the 5’ UTR of viral RNA (Pelletier and Sonenberg 1988; Jang et al. 1988), exhibiting complex structures and capable of recruiting translation machinery within host cells. There is a more detailed review that discusses how viral RNA structures manipulate RNA translation (Jaafar and Kieft 2019). Different RNA virus families are characterized by distinct IRES elements which have been reviewed by other reviews (Peng et al. 2023; Niepmann and Gerresheim 2020; Nakashima and Uchiumi 2009; Lozano and Martínez-Salas 2015). One study uses RNase V1 probing to determine the 5’ UTR in SARS-CoV-2 and found that there is a highly stable four-way junction in this region, which may be involved in translation initiation (Miao et al. 2021). Additionally, structural elements situated in the 3’ UTR influence translation processes as well, which is a topic thoroughly reviewed elsewhere (Rasekhian et al. 2021).

Virus replication is also regulated by RNA secondary structure. Liu et al*.* found that disruption of the pseudoknots structures, achieved through IRES point mutations in the of Senecavirus A (SVA), inhibits virus replication rather than its translation (Liu et al. 2021a; Liu et al. 2021b). They identified other essential structural elements (stem II and stem Ib) in SVA that are crucial for virus replications. Enterovirus has a structural element in the 5’ UTR called the Cloverleaf (CL) structure, which provides platforms for 3CD and PCBP2 recruiting to promote viral replication (Das et al. 2023). Some reviews can provide overall perspectives on how RNA structure can regulate viral replications (Malone et al. 2022; Szczesniak, et al. 2023).

RNA structure probing methods, such as SHAPE-Map(Huston et al. 2021), MarathonRT (Guo et al. 2022), DMS-MaPseq (Lan et al. 2022), icSHAPE (Sun et al. 2021a; Li et al. 2018), etc., have been instrumental in determining the genome-wide in vivo structures of viruses like SARS-CoV-2 and ZIKV. These methods have paved the way for understanding the virus RNA structurome, allowing the discovery of more drug-targetable sites. Despite the comprehensive structural-function map of SARS-CoV-2 generated by Sun et al*.* (Sun et al. 2021a), Li et al*.* also employed icSHAPE and PARIS techniques to analyze the viral genome structure of ZIKV (Li et al. 2018). They identified key structural elements that are crucial for viral infestation of cells and constructed structure–function maps. SHAPE-Map was also used to detect interactions between RNA and small molecules, which has provided us with a new way for antiviral drug screening (Martin et al. 2019). RNA viruses exemplify the close interplay between the structure and function of RNA. Utilizing RNA secondary structure probing methods, researchers can uncover the in vivo secondary structure of RNA and RNA-RNA interactions (Lu et al. 2016). This approach facilitates the identification of crucial structural elements, enables the analysis of structure–function relationships, and reveals potential RNA drug targets. Therefore, when combined with an in-depth analysis of RNA–protein interactions, it not only establishes the groundwork for a comprehensive understanding of these relationships but also extends the pool of potential targets for drug screening.

## Conclusion

Precise investigation of RNA structure is vital in advancing our understanding of life processes. Recent years have witnessed rapid advancements in RNA secondary structure probing techniques, with many of them achieving single-nucleotide resolution. These technological strides have led to the development of RNA Structurome research, also providing comprehensive insights into RNA secondary structures. These insights have significantly contributed to our understanding of diverse cellular regulatory processes, including epigenetic regulation, riboswitch mechanisms, RNA splicing, and RNA stability. Meanwhile, they have facilitated the creation of therapeutic tools for tackling human diseases. Despite their therapeutic applications, RNA structure probing methods also offer a promising avenue for exploring the mechanisms of human diseases, potentially providing the key to overcoming existing research constraints and obtaining the in-depth information necessary for a deeper understanding of disease mechanisms.

Remarkably, RNA structural research has entered a new era, in which advanced computational and experimental methods are used to study RNA structurome. This enables the development of various clinical tools and opens new perspectives for understanding disease mechanisms and developing more efficient drugs. However, there still exists hurdles in both determining the interested RNA structures and the applications in human diseases. First, the existing RNA secondary probing methods inadequately cover all positions in the transcriptome or genome, resulting in certain positions within a specific transcript lacking corresponding icSHAPE scores. This may lead to an unrealistic RNA secondary structure in these uncertain positions, which can hinder scientists from studying the related RNA and disease mechanisms. In the future, more sensitive and precise probing methods need to be developed by changing the experimental conditions or improving the efficiency of enzymes. Second, existing tools developed for clinical treatment are rarely applied due to potential side effects, such as off-target effects caused by inducible CRISPR/Cas9 systems based on riboswitches. Scientists should develop more effective ways to evaluate these tools and improve their safety. Third, deep learning models have certain limitations, notably issues like overfitting and inaccurate predictions. These challenges can arise due to several factors, including overcomplicated parameterization during model establishment and the presence of high background noise in the training datasets. Additionally, the accuracy of prediction models is often closely tied to the quantity of training data, making precise secondary structure predictions for low-abundant RNAs particularly challenging. Overall, addressing these limitations requires researchers to optimize network architectures and enhance the quality of experimental datasets.

## Data Availability

No datasets were generated or analyzed during the current study.
